# Gas Gangrene: Its Course and Treatment

**Published:** 1917-12

**Authors:** 


### PATHOLOGY AND DIAGNOSIS

**Gas Gangrene : Its Course and Treatment.** By KENNETH TAYLOR, Pathologist, American Ambulance Hospital, Paris, and Director of the Laboratories of the Robert Walton Goelet Research Fund. Abs, from the *Johns Hopkins Hospital Bulletin*, Vol XXVII, N° 308, Oct., 1916.

The author defines " gaseous gangrene " as " the death of an extensive mass of muscle due to the mechanical action of gas produced from a local focus by saprophytic bacteria ".

Muscle, he believes, is the tissue chiefly involved, because the substances from which the gas is formed are chiefly the carbohydrate-containing tissues. The organism is, with rare exceptions, B. aerogenes capsulatus welchii (B. perfringens).

The first stage of gas infection the author describes as " *dormant* ". This condition is present in the majority of wounds. The organism has heen found present in 70 o/o of all wounds examined bacteriologically at a general military hospital. The bacteria are to be found in the dead muscle and gas is sometimes evident in the depths of the wound.

The second stage, that of " *gaseous distension* ", is marked by a gaseous infiltration of healthy tissue. Retention of the gas causes sustained pressure, The rapid increase of this intramuscular pressure may quickly deprive the tissues of blood until they appear to be wrung dry of fluids. At this point the condition of gangrene supervenes.

The " *explosive stage* " may then follow, in which there is rapid progress of the infection due to the invasion of the gangrenous muscle by the bacilli.

The " *stage of systemic toxemia* " may accompany or follow rapidly upon the preceding one. Collapse and death usually result. Occasionally a stage of terminal bacteriemia is reached.

Evidently the conditions which determine the extension of the process from stage 1 to stage 2 are the most important from a therapeutic point of view. After gaseous distension has developed the problem becomes much more complicated. The author reviews the various explanations of this change :

1. It is contended that several different organisms may produce the disease. T. believes, on the other hand, that there is but one distinct species — B. aerogenes capsulatus — responsible for nearly all cases of gas gangrene. B. edematis maligni is the only other gas producing organism that may cause extensive lesions in the muscles, but this bacillus rarely, if ever, gives rise to extensive gaseous phlegmons. It is, however, frequently found in the wound itself. The frequent occurrence of subcutaneous edema, the author believes, is due to the obstruction of the deep lymphatics and veins by intramuscular pressure and not to this organism.

2. T. does not believe that the different forms of the disease are due to variations in the virulence of the organism, because the various strains of B. aerogenes capsulatus appear almost equally virulent for animals, even when taken from human cases of varying severity.

3. T. does not believe that the invasion of the blood by the organism can be responsible for the malignant type of the disease, because the bacillus is rarely to be found in the blood before death.

4. As to the theory that the absorption of soluble toxic products of the bacteria breaks down the natural immunity of the patient, T. contends that the exotoxin from the organism is of only small toxicity. Symptoms of toxicity from a local limited focus are slight. Only when large masses of muscle are destroyed by the gas does toxemia occur. " Then the putrefactive and autolytic colliquative necrosis induces rapid and profound toxic symptoms. The real action of the toxic principle is... that of converting healthy muscle tissue adjacent to the wound into a favorable medium for growth and gas production by the bacteria. "

5. Against the theory that the progress of the disease is due to the failure of active immunity, T. contends that the spread of the infection is too rapid to allow time for the production of antibodies. Besides, no prompt systemic reaction is likely to result because bacterial activity is chiefly limited to detached and necrotic tissues. No immune reaction could be demonstrated in the sera of men or animals that had recovered from the infection or that had received prophylactic gas-bacillus vaccine. The bacillus is rarely phagocytosed in the wound. No increased resistance could be demonstrated in animals that had recovered from a previous infection. Guinea-pigs show no variation in individual susceptibility to experimental inoculation.

6. The spread of the infection is frequently charged to injury and thrombosis of important blood vessels. T. has found thrombosis in autopsy in only a few cases (3 out of 19), and believes it to have been the result rather than the cause of gaseous distension. Ligature of the main artery to a limb, however, may produce the death of tissues in much the same way as gaseous distension.

7. T. has shown that the gas itself is not toxic.

8. To the contention that symbiosis of the gas bacillus with other organisms is responsible for a malignant infection, T. replies that there is no constant similarity between the flora of the various cases of gas gangrene.

Excluding these considerations as probable causes for the progression of the disease, T. looks for an explanation in the conditions of the wound itself. Two factors seem to be necessary : (1) A continuous production of gas in the wound; (2) Sustained pressure within a muscle mass, following its infiltration and distension by the gas formed.

1. An adequate supply of dead muscle is of first importance in affording the saprophyte an anaerobic medium favorable for its multiplication and the production of gas and toxin. Hence the importance of injury by shell and shrapnel with a high velocity and an explosive effect upon the tissues, in addition to tearing, fragmentation, and the destruction of the blood supply. Ragged bone splinters are even more effective in producing such conditions.

Almost invariably the activity of the bacteria is confined to the muscle. It is rarely active in the subcutaneous tissues, and then usually in the scalp and scrotum where the skin is rich in muscle tissues. Subcutaneous crepitus is merely evidence of gas that has escaped from the muscle. Only after exudation of the products of muscle degeneration into the subcutaneous tisues is the organism found growing and producing gas in this location.

2. "Sustained pressure within a muscle mass depends on retention by intact muscle sheaths of the gas produced, and by the occlusion of the avenues of escape, due to the local swelling of the muscle fibers in response to the inflamatory reaction. The structure of muscle permits an easy infiltration of gas between and parallel to the fiber bundles. The same arragement of fibers makes its escape correspondingly difficult except in longitudinal direction, and this outlet is frequently blocked by the bulging of fibers into the wound. This blocking of the avenues of escape for the gas is favored by various conditions. It is especially apt to occur when fracture of a long bone is present. This deprives the muscles of their splint, which would otherwise allow the longitudinal contraction of the cut fibers to tend to keep the wound open. When a fracture occurs, the contraction of the muscles closes the wound more firmly by allowing of lateral bulging. It follows, therefore, that an infected fracture is more easily drained of gas and pus when held in extension than before traction is applied. "

(This abstract is concluded under "Treatment", p. 126.)

In the *Lancet* for Dec., 23, 1916, T. adds to these observations an account of " Two Fatal Cases of Metastatic Gas Gangrene ". In one of these metastatic gangrene developed in the gluteal muscles five days after amputation of the right arm and excision of shoulder muscle for gaseous gangrene. The region of the operation indicated a complete arrest of the infection. After the treatment of the arm and shoulder, the patient had been placed in a sitting posture, and the pressure thus brought to bear upon the muscles, at the site of thesecondary infection, was, T. believes, the cause of this phenomenon.

Similarly in the second case, the patient had rested on the left side because of an injury to the right buttock. The wound was six days old. The right gluteal muscles were widely opened. The general condition grew worse, and it was found that gaseous gangrene had developed in the *left thigh.* Autopsy showed no communication whatever between the wound of the right gluteal muscles and the infected tissues in the left thigh or left gluteal region. Intervening muscles and subcutaneous tissues were carefully examined, but there was no indication of infection. B. aerogenes capsulatus was found in the two infected regions. Culture of the heart's blood, however, was sterile.

The author compares the cases and concludes :

" 1. That blood invasion may occur during the life of the patient but is probably of only a short duration.

" 2. That the vitality of a muscle mass may be so lowered by continuous subjection to pressure and the resultant interference with its circulation that it may become a site for the fixation and activity of the bacilli following the temporary invasion of the blood.

" 3. That it is advisable, in the case of a patient who has a wound infected by the gas bacillus, to make a careful examination of the entire body and not limit the attention to the wound.

" 4. That it is dangerous to allow a patient suffering from a wound infected by the gas bacillus to maintain a fixed position in bed. "

